# Protein Kinase D3 (PKD3) Requires Hsp90 for Stability and Promotion of Prostate Cancer Cell Migration

**DOI:** 10.3390/cells12020212

**Published:** 2023-01-04

**Authors:** Attila Varga, Minh Tu Nguyen, Kinga Pénzes, Bence Bátai, Pál Gyulavári, Bianka Gurbi, József Murányi, Péter Csermely, Miklós Csala, Tibor Vántus, Csaba Sőti

**Affiliations:** 1 Department of Molecular Biology, Semmelweis University, 1094 Budapest, Hungary; 2 MTA-SE Pathobiochemistry Research Group, Semmelweis University, 1094 Budapest, Hungary; 3 Institute of Medical Microbiology, Semmelweis University, 1089 Budapest, Hungary; 4 HCEMM-SU Molecular Oncohematology Research Group, Department of Pathology and Experimental Cancer Research, Semmelweis University, 1085 Budapest, Hungary; 5 IQVIA Hungary, 1117 Budapest, Hungary

**Keywords:** protein kinase D, heat shock protein 90, prostate cancer, migration, metastasis

## Abstract

Prostate cancer metastasis is a significant cause of mortality in men. PKD3 facilitates tumor growth and metastasis, however, its regulation is largely unclear. The Hsp90 chaperone stabilizes an array of signaling client proteins, thus is an enabler of the malignant phenotype. Here, using different prostate cancer cell lines, we report that Hsp90 ensures PKD3 conformational stability and function to promote cancer cell migration. We found that pharmacological inhibition of either PKDs or Hsp90 dose-dependently abrogated the migration of DU145 and PC3 metastatic prostate cancer cells. Hsp90 inhibition by ganetespib caused a dose-dependent depletion of PKD2, PKD3, and Akt, which are all involved in metastasis formation. Proximity ligation assay and immunoprecipitation experiments demonstrated a physical interaction between Hsp90 and PKD3. Inhibition of the chaperone–client interaction induced misfolding and proteasomal degradation of PKD3. PKD3 siRNA combined with ganetespib treatment demonstrated a specific involvement of PKD3 in DU145 and PC3 cell migration, which was entirely dependent on Hsp90. Finally, ectopic expression of PKD3 enhanced migration of non-metastatic LNCaP cells in an Hsp90-dependent manner. Altogether, our findings identify PKD3 as an Hsp90 client and uncover a potential mechanism of Hsp90 in prostate cancer metastasis. The molecular interaction revealed here may regulate other biological and pathological functions.

## 1. Introduction

Prostate cancer is the most frequently diagnosed tumor in men. Androgen deprivation therapy elicits a favorable initial response [1]. However, a frequent outcome is the manifestation of an androgen-independent metastatic castration-resistant prostate cancer [2]. Distant metastases due to the highly invasive nature at this stage are the major cause of death [3]. The genetic changes and the molecular mechanisms underlying the development of metastatic castration-resistant disease are still not entirely clear. The increased invasive potential of these tumors was connected to alterations in androgen receptor (AR), phosphoinositide 3-kinase (PI3K), and WNT signaling pathways [4]. Besides targeting these pathways, a better understanding of other migration-promoting signals and inhibition/prevention of invasion is essential for developing effective therapies against prostate cancer.

The serine/threonine protein kinase D (PKD) family belongs to the calcium/calmodulin-dependent kinase superfamily and has three identified members: PKD1, PKD2, and PKD3 [5,6,7], which share significant structural homology. The N-terminal regulatory domain consists of two cysteine-rich diacylglycerol-binding C1 domains and an auto-inhibitory pleckstrin homology domain. The C-terminal part contains the catalytic domain and a PDZ-domain, which is absent in PKD3 [8]. Various stimuli, including growth factors, hormones, diacylglycerol, and oxidative stress, activate PKD enzymes [9], which indicates that PKD isoforms regulate diverse cellular processes including cell growth, proliferation, cell migration/invasion, apoptosis, and epithelial-to-mesenchymal transition (EMT) [9].

PKD3 supports tumor growth and metastasis in several tumor types, such as prostate cancer [10,11]. PKD3 was reported to regulate tumor growth and survival through activation of Akt and extracellular signal-regulated kinase 1/2 (Erk1/2) and through upregulation of lipogenesis via sterol regulatory element binding protein 1 (SREBP1) [12,13]. PKD3 also stimulates secretion of multiple tumor-promoting factors including matrix metalloproteinase-9 (MMP-9), interleukin-6 and 8, and growth regulated alpha protein (GROα) [14]. PKD3 together with PKD2 initiate prostate cancer cell migration by modulating urokinase-type plasminogen activator (uPA) expression and activation in a nuclear factor kappa B (NF-κB) and histone deacetylase 1 (HDAC1)-mediated manner [15]. Other studies showed that genetic inhibition of PKD3 and administration of PKD inhibitors efficiently diminished prostate cancer cell proliferation and migration in vitro and in vivo [14]. Data from human prostate cancers and tumor cell lines of this origin indicate that PKD3 expression and nuclear localization correlate with tumor grade: specifically, androgen-independent metastatic tumors exhibit high PKD3 protein expression [12,13]. The above findings suggest that PKD3 is an important regulator of androgen-independent prostate cancer migration, but the molecular requirements of its function are unclear.

The molecular chaperone Hsp90 is an essential, conserved cytosolic heat-shock protein, which emerged as an important ’non-oncogenic’ determinant of malignant transformation [16,17]. Its N-terminal ATP-binding and middle-C-terminal domains work in a coordinated manner to ensure conformational stability to its substrate proteins called ’clients’ [18]. The several hundred clients are all thermodynamically unstable multidomain signaling proteins. For instance, Hsp90 stabilizes ~60% of the human kinome and various steroid receptors [18,19]. Its clientele also includes various PKC isoforms, PKD2, and the androgen receptor [20,21]. An up-to-date list of clients is found at www.picard.ch/downloads/Hsp90interactors.pdf (accessed on 12 December 2022).

Although Hsp90 is indispensable in non-transformed cells, the intensified flux of signaling pathways combined with homeostatic disturbances largely increases the tumor cells’ dependence on Hsp90 and their vulnerability to Hsp90 inhibitors [22,23]. More than 400 Hsp90 clients are involved in cancer signaling [23]. The majority of Hsp90 inhibitors, such as the chemotherapeutic ganetespib, target the N-terminal ATP-binding site [24,25,26]. This in turn leads to destabilization and proteasomal degradation of the clients, thus compromising cancer signaling, survival, and invasion [22,23]. Hsp90 inhibitors are tested in clinical trials against various tumors including androgen-dependent and -independent prostate cancers [27]. Hsp90 also appears to promote prostate cancer metastasis by various, although not entirely elucidated, mechanisms [27,28,29,30]. In this study, we show that Hsp90 stabilizes PKD3 in different prostate cancer cell models and this interaction facilitates tumor cell migration.

## 2. Materials and Methods

### 2.1. Materials

Reagents for cell cultures were from Gibco (Waltham, MA, USA). Ganetespib, CRT0066101, and bortezomib were from Selleckchem (Houston, TX, USA). Anti-PKD3 and non-targeting siRNAs and transfection reagent DharmaFECT were from Dharmacon (Lafayette, CO, USA). pEGFP-N1-PKD3 and pEGFP-C1 plasmids were kind gifts from Ugo Moens (UiT The Arctic University of Norway, Tromso, Norway) or Adrienn Borsy (Institute of Enzymology, Research Center of Natural Sciences, Budapest, Hungary). If not otherwise stated, all other reagents were from Merck (Darmstadt, Germany).

### 2.2. Cell Culture

The prostate cancer cell lines DU145, PC3, and LNCaP were from ATCC. LNCaP and PC3 cells were grown in RPMI-1640 and DU145 cells in Eagle’s Minimum Essential Medium, supplemented with 10% FBS in 5% CO_2_ at 37 °C. The cell lines were tested for mycoplasma contamination using Hoechst staining and were authenticated performing STR profile analysis (Eurofins Scientific, Budapest, Hungary).

### 2.3. Cell Migration

Cells were harvested by trypsinization, centrifuged with 150× *g* for 4 min, and resuspended in serum-free media. 10^5^ (DU145, PC3) or 2 × 10^5^ (LNCaP) cells were added into 8-µm ThinCert^TM^ culture inserts (Greiner Bio-One, Kremsmünster, Austria). Bottom wells contained culture media with 10% FBS as chemoattractant. After incubation for the indicated times at 37 °C, cells were fixed in methanol and stained with 0.2% crystal-violet solution. Non-migrating cells were removed by cotton swabs from the inner membrane of the insert. Five photos from each insert were taken using Olympus IX73 inverted microscope. Migrated cell numbers were determined by pixel number, using GNU Image Manipulation Program (GIMP).

### 2.4. Gene Transfection

LNCaP cells were seeded into 6-well plates at 5 × 10^5^ cells/well and incubated overnight. Then, cells were transfected with pEGFP-C1 or pEGFP-N1-PKD3 plasmids, respectively, using Lipofectamine LTX (Thermo Fisher Scientific, Waltham, MA, USA) and Opti-MEM medium (Gibco, Waltham, MA, USA) for 24 h. Then, cells were further incubated for 24 h at 37 °C in RPMI medium with FBS.

### 2.5. Cell Lysis

Cells were washed with ice-cold PBS and lysed in RIPA buffer (50 mM Tris (pH 7.4), 150 mM NaCl, 1% NP-40, 0.5% sodium-deoxycholate, 0.1% sodium-dodecyl-sulphate, 2 mM EDTA, 2 mM EGTA, 1 mM dithiothreitol) freshly complemented with phosphatase and protease inhibitor cocktail (Merck, Darmstadt, Germany) for 30 min on ice. Lysates were centrifuged at 13,000× *g* at 4 °C for 15 min. Protein concentration was determined by the Bradford method (Bio-Rad, Hercules, CA, USA). Aggregates in the pellet were solubilized in urea-containing lysis buffer (2% SDS, 6 M urea, 30 mM Tris, pH 7.6) on ice for 30 min, and then centrifuged with 13,000× *g* at 4 °C for 15 min. Supernatants were used for Western blotting.

### 2.6. Western Blot

Equal amounts of protein were subjected to SDS-PAGE and then electrotransferred to polyvinylidene-difluoride (PVDF) membranes. Mouse monoclonal anti-pan-Akt, rabbit polyclonal anti-β-actin, rabbit monoclonal anti-pSer536-NF-κB-p65, anti-NF-κB-p65, anti-PKD2, rabbit monoclonal anti-PKD3, and HRP-conjugated secondary horse anti-mouse and goat anti-rabbit IgG antibodies were from Cell Signaling Technologies (Danvers, MA, USA). Mouse monoclonal anti-Hsp90 antibody was from Institute of Immunology Ltd. (Tokyo, Japan). Membranes were probed with primary antibodies at 4 °C overnight and HRP-conjugated secondary antibody for 1 h at room temperature. Bands were visualized by Enhanced Chemiluminescence (Perkin Elmer, Buckinghamshire, UK) and quantified by ImageJ (National Institute of Health, Bethesda, MD, USA).

### 2.7. Proximity Ligation (PLA) Assay

DU145 and PC3 (3 × 10^4^ cells/well) were seeded into 96-well µ-plates (Ibidi GmbH, Grafelfing, Germany) and allowed to attach overnight. Then, cells were washed with PBS, fixed with paraformaldehyde and permeabilized with 0.1% Triton-X (-100) containing PBS. Wells were blocked with PLA Blocking solution (Sigma Aldrich, St. Louis, MO, USA) for 1 h, then primary antibodies diluted in PLA antibody diluent solution (Sigma Aldrich, St. Louis, MO, USA) were added and incubated overnight at 4 °C. Rabbit polyclonal anti-Akt1 antibody was from Sigma–Aldrich, mouse monoclonal anti-Hsp90 from Institute of Immunology Ltd. (Tokyo, Japan), and rabbit polyclonal anti-PKD2 and rabbit polyclonal anti-PKD3 antibodies from Atlas Antibodies (Bromma, Sweden). Then, the experiment was carried out according to the manufacturer’s instructions (Sigma Aldrich, St. Louis, MO, USA).

### 2.8. Co-Immunoprecipitation (Co-IP)

Cells were washed and scraped in ice cold PBS and lysed in IP buffer (50 mM Tris, 2 mM EDTA, 100 mM NaCl, 1 mM Na_3_VO_4_, 1% NP40, freshly complemented with protease inhibitor cocktail (Merck, Darmstadt, Germany), pH 7.6). PKD3 was immunoprecipitated from 1000 µg total protein by a mouse monoclonal anti-PKD3 antibody (Santa Cruz Biotechnology, Inc., Dallas, TX, USA). Pellets were washed five times with IP buffer and analyzed by SDS–PAGE and immunoblotting.

### 2.9. Statistical Analysis

Statistical analysis was performed by one-way ANOVA with Dunett’s multiple comparison test, multiple t-tests and two-way ANOVA with Tukey’s multiple comparison test using GraphPad Prism 9 software (San Diego, CA, USA). Experiments were repeated at least twice. Data were expressed as means ± SEM. Statistical levels of significance were indicated as follows: * *p* < 0.05; ** *p* < 0.01; *** *p* < 0.001; **** *p* < 0.0001.

## 3. Results

### 3.1. Inhibition of Hsp90 or PKD Arrests Androgen-Independent Prostate Cancer Cell Migration

**Figure 1:**
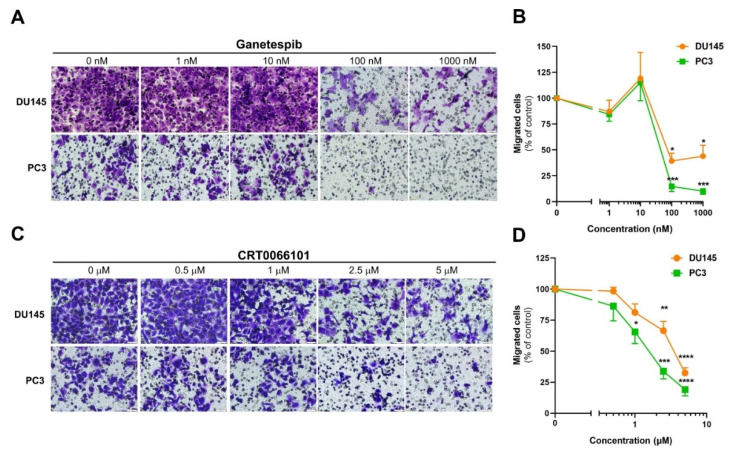
Inhibition of Hsp90 or PKD attenuates the migration of androgen-independent DU145 and PC3 prostate cancer cells. Cells were allowed to migrate for 22 h in the presence of the indicated doses of the inhibitors. Representative microscope images of ganetespib (**A**) and CRT0066101 (**C**) treatments, respectively. The scale bars represent 50 µm. Quantification of the results of ganetespib (**B**) and CRT0066101 treatment, respectively (**D**). All data are mean ± SEM values of 3 independent experiments. * *p* < 0.05, ** *p* < 0.01, *** *p* < 0.001, **** *p* < 0.0001 (two-way ANOVA with Tukey’s multiple comparison test).

First, we tested the role of Hsp90 in prostate cancer cell migration employing its specific inhibitor ganetespib (GS) [25]. We used the well-established DU145 and PC3 model cell lines, originating from highly invasive androgen-independent metastatic tumors. We incubated the cells in culture inserts with various concentrations of GS for 22 h and assessed cell migration after 22 h. After an initial, non-significant increase characteristic to the hormetic effect of low dose Hsp90 inhibition [31], GS exerted a potent anti-migratory effect on both cells at 100 and 1000 nM concentration (Figure 1A,B), which is consistent with the effective concentration range of GS reported in several tumor models. Maximum inhibition was reached at 100 nM (Figure 1B). GS treatment exerted a more potent, almost complete anti-migratory effect on PC3 cells, suggesting that Hsp90-dependent mechanisms play an indispensable role at this genetic background.

Next, we asked whether PKD inhibition affects cell migration in our conditions. In accordance with previous findings [11], the pan-PKD inhibitor CRT0066101 robustly diminished migration of both cell lines in a concentration-dependent manner (Figure 1C,D). Similar to Hsp90 inhibition, PC3 cells displayed a more pronounced response to the inhibitor, showing a maximal extent comparable to GS treatment (Figure 1D). The stronger inhibition might be due to higher PKD3 and PKD2 protein levels in PC3 compared to DU145 cells (Figure S1). Alternatively, CRT0066101 may inhibit other kinases involved in cell motility. Nevertheless, these findings show a critical requirement for both Hsp90 and PKDs (and related kinases) for prostate cancer cell migration, which is consistent with previous data showing roles for PKD3, PKD2, and Hsp90 in prostate cell migration and tumor metastasis [10,11,28,29].

### 3.2. Hsp90 Inhibition Decreases PKD3 Protein Level in Prostate Cancer Cells

**Figure 2:**
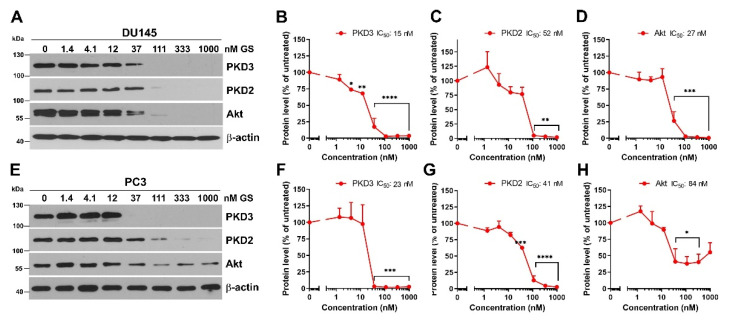
Hsp90 inhibition depletes PKD3 protein in prostate cancer cells. Western blots of lysates from DU145 (**A**) and PC3 (**E**) cells treated with GS for 48 h. Densitometric analysis and IC_50_ calculation (mean ± SEM) from DU145 (**B**–**D**) and PC3 (**F**–**H**) cells from three (or two, panels (**C**,**D**,**H**)) independent experiments. * *p* < 0.05, ** *p* < 0.01, *** *p* < 0.001, **** *p* < 0.0001, (two-way ANOVA with Tukey’s multiple comparison test). Western blot images are representatives of three (or two, panels (**C**,**D**,**H**)) independent experiments.

Since PKD2 has been identified as an Hsp90 client [11,20], we hypothesized that Hsp90 might promote prostate cancer cell migration via PKD3. Exposure of cells to Hsp90 inhibitors results in the depletion of client proteins [22,23]. To address whether PKD3 is an Hsp90 client, we treated both cell lines with a range of GS doses and estimated PKD3 levels after 48 h. We observed a significant decrease in PKD3 protein level in a dose-dependent manner in both cancer cell lines (Figure 2A,E). The IC_50_ values were almost identical (DU145: 15 nM, PC3: 23 nM), showing a comparable dependence of PKD3 protein on Hsp90 in both cells (Figure 2B,F). GS treatment effectively reduced the Hsp90 client PKD2 level in a dose-dependent manner, yielding comparable, although slightly higher IC_50_ values than those of PKD3 (DU145: 52 nM, PC3: 41 nM), confirming the earlier findings (Figure 2C,G) [20]. We monitored the protein level of another well-known Hsp90 client protein Akt, which is also involved in prostate cancer downstream of PKD3 [12,32]. GS treatment completely diminished Akt protein level in DU145 cells, whereas the effect was partial and the IC_50_ value was higher in PC3 cells (27 nM vs. 84 nM) (Figure 2D,H). These results confirm and extend the original report [20] on Hsp90–PKD2 interaction and suggest that beyond PKD2, Hsp90 also stabilizes PKD3 in two independent prostate cancer cell lines.

### 3.3. A Physical Interaction with Hsp90 Ensures PKD3 Conformational Stability

**Figure 3:**
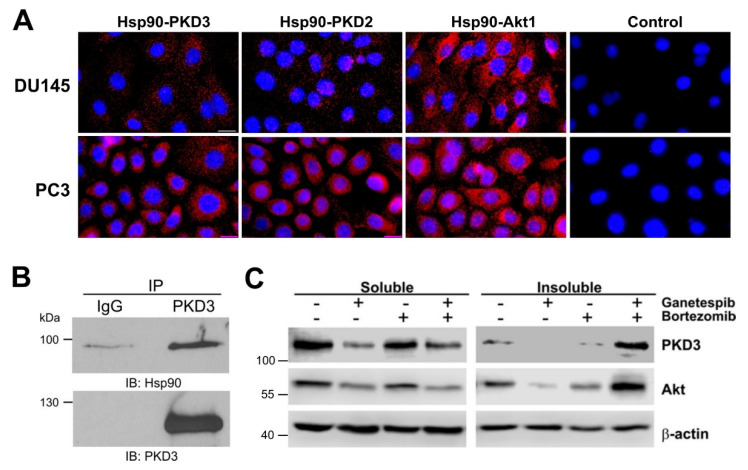
Hsp90 directly interacts with PKD3 in DU145 and PC3 cells. (**A**) Cells were probed with the indicated antibodies to detect direct protein-protein interactions in situ applying PLA. Controls contained anti-mouse and anti-rabbit secondary antibodies. Scale bars represent 20 µm. (**B**) PKD3 was immunoprecipitated from PC3 cells and co-immunoprecipitated Hsp90 was analyzed by Western blot. (**C**) Hsp90 inhibition leads to the proteasomal degradation of PKD3. DU145 cells were treated with ganetespib or/and bortezomib as indicated for 24 h and the soluble and insoluble lysates were analyzed by Western blot. Images are representatives of three independent experiments.

Next, we employed Proximity Ligation Assay (PLA) to investigate whether a direct, physical connection exists between PKD3 and Hsp90. PLA is a sensitive method, which allows detection of direct protein-protein interactions at 40 nm distance between endogenous proteins in situ [33]. During the assay, the two target proteins in the cells are stained with primary antibodies from different species (i.e., rabbit/mouse), followed by staining with secondary antibodies conjugated with a unique DNA strand. If the proteins of interest interact, DNA strands hybridize and make circular DNA, which can be amplified and labeled with fluorescent oligonucleotides [33]. We observed a specific fluorescence in the presence, but not in the absence, of the PKD3 antibody in both cell types (cf. Figure 3A first and fourth micrographs), suggesting a molecular proximity of PKD3 and Hsp90. The PKD2 antibody showed an interaction with Hsp90, similar to that detected with PKD3, in both cell types. Interestingly, PC3 cells exhibited much stronger signals for both PKD3 and PKD2, compared to DU145 cells. As experiments on PC3 and DU145 cells were performed separately, it prevented us to make a quantitative comparison. However, this result appears consistent with much higher PKD3 and PKD2 protein levels in PC3 vs. DU145 cells (cf. Figure 3A and Figure S1). Hsp90 is a chaperone for all Akt protein kinase isoforms [34]. Indeed, an Akt1 antibody also exhibited an even higher fluorescence signal compared to PKD2 and 3, perhaps due to its abundance. We independently demonstrated the Hsp90–PKD3 interaction in PC3 cells by co-immunoprecipitation (Figure 3B). Altogether, these results confirm the previously reported PKD2–Hsp90 interaction and demonstrate a direct interaction between PKD3 and Hsp90.

In response to conformational destabilization, Hsp90 clients are degraded by the proteasome [18]. To address this possibility for PKD3, we treated the cells simultaneously with GS and the proteasome inhibitor bortezomib. After separation of the soluble and insoluble fractions by sedimentation, we solubilized the insoluble proteins using SDS and urea. Bortezomib treatment alone changed neither PKD3 nor Akt protein level. However, in combination with GS, both PKD3 and Akt proteins appeared in the insoluble fraction, indicating that misfolded PKD3 is degraded by the proteasome (Figure 3C). Using the PLA assay, we found that a short term, 4-h GS treatment did not decrease the interaction of PKD3 with Hsp90 in situ, perhaps the destabilized client is not dissociated but remains in a loose complex with Hsp90 while channeled to the UPS for degradation (Figure S3A,B). We obtained similar result for PKD2 (Figure S3A,C), which indicates similar interactions with the two PKD isoforms with Hsp90. Altogether, these results clearly establish a requirement for Hsp90 in the conformational stabilization of PKD3.

### 3.4. The Hsp90–PKD3 Interaction Is Required for PKD3-Dependent Phosphorylation of p65

**Figure 4:**
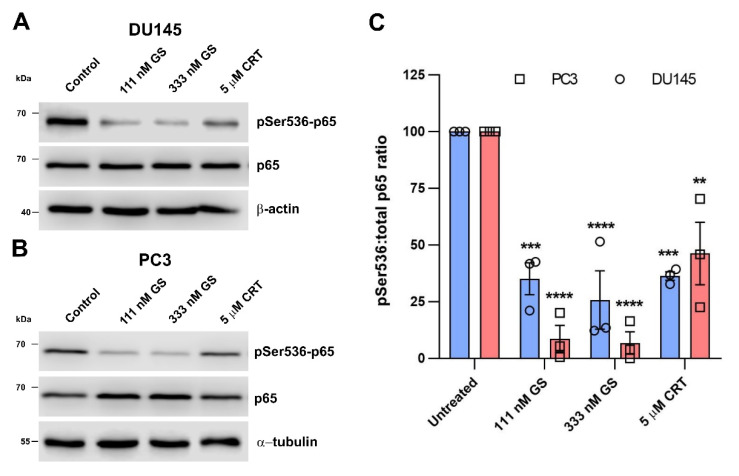
Hsp90 and PKD inhibition impedes Ser536 phosphorylation of the NF-κB subunit p65. Representative Western blots of lysates from DU145 (**A**) and PC3 (**B**) cells treated with 111 or 333 nM GS or 5 μM CRT0066101, respectively, for 4 h. (**C**) Densitometric analysis (mean ± SEM) from three independent experiments. ** *p* < 0.01, *** *p* < 0.001, **** *p* < 0.0001, (two-way ANOVA with Tukey’s multiple comparison test).

We hypothesized that the loss of PKD3 functional conformation by Hsp90 inhibition affects PKD3-dependent signaling events involved in prostate cancer invasion. PKD3 together with PKD2 promotes prostate cancer cell migration through the expression and activation of urokinase-type plasminogen activator (uPA) via the NF-κB pathway [15]. Specifically, PKD3 is indispensable for the Ser536 phosphorylation of the NF-κB p65 subunit. Consistent with our hypothesis, both a 4-h GS and CRT0066101 treatment, respectively, reduced the Ser536 phosphorylation of p65 in both DU145 and PC3 cells (Figure 4A–C). We conclude that the structural destabilization of PKD3 compromises its function and downstream signaling.

### 3.5. Hsp90 Chaperones PKD3-Mediated Prostate Cancer Cell Migration

**Figure 5:**
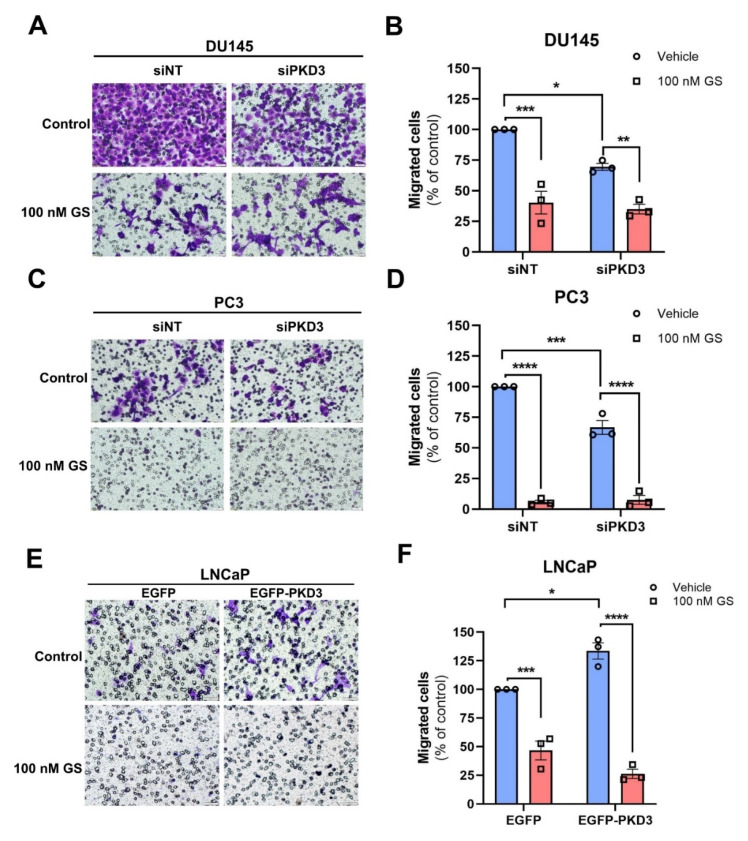
Stabilization of PKD3 by Hsp90 is essential for prostate cancer cell migration. Representative microscope images of DU145 (**A**) and PC3 (**C**) cell migration in response to PKD3 gene silencing and/or ganetespib (GS) treatment. Quantification of the results (mean ± SEM) obtained with DU145 (**B**) and PC3 (**D**) cells from panels (**A**,**C**). Cells were PKD3 silenced by specific siRNAs for 48 h, then cell motility was investigated in the absence or presence of 100 nM GS for 22 h. (**E**) Representative microscope image of LNCaP cell migration in response to PKD3 gene transfection and/or GS treatments. Quantification of the results (mean ± SEM) from panel (**F**). LNCaP cells were transfected with EGFP or EGFP–PKD3 expressing vectors for 24 h, then cell migration was investigated in the absence or presence of GS for 24 h. Scale bars represent 50 µm. All data are from 3 independent experiments. * *p* < 0.05, ** *p* < 0.01, *** *p* < 0.001, **** *p* < 0.0001, (one-way ANOVA with Dunett’s multiple comparison test).

PKD3 is overexpressed in many cancers, which has been demonstrated to contribute to their invasive nature [10,14,15]. Hsp90 has been reported to stabilize other factors that play a role in cell migration [35], also in prostate tumors [14,15,29]. Therefore, we set out to directly estimate the contribution of the Hsp90–PKD3 interaction as well as the individual, specific impact of PKD3 on prostate cancer cell migration. To this end, we successfully knocked down PKD3 by an anti-PKD3 siRNA in both cell types, which did not affect PKD2 (Figure S2A,B). PKD3 depletion reduced cell migration by 33% in DU145 and by 39% in PC3 cells (Figure 5A–D). The slightly higher contribution of PKD3 vs. markedly higher protein levels in PC3 cells suggests that PKD3 levels are already sufficient in DU145 to promote cell migration. We confirmed this assumption by PKD3 overexpression. DU145 cells could not be transfected; however, GFP-fused PKD3 was overexpressed in PC3 cells and depleted upon GS treatment, hence overexpressed PKD3 preserved its dependence on Hsp90 (Figure S4A). Increased PKD3 dosage did not significantly augment PC3 cell migration (Figure S4B). Hsp90 inhibition resulted in 61% and 93% reduction in migration of DU145 and PC3 cells, respectively, suggesting a higher reliance of PC3 migration on Hsp90-dependent processes (Figure 5A–D). Importantly, PKD3 silencing did not augment the effect of Hsp90 inhibition. These results indicate that in both cells, PKD3 entirely depends on Hsp90 for executing its cell migratory functions.

The androgen-dependent weakly metastatic cell line LNCaP exhibits negligible PKD3 protein expression (Figure S1). Yet, a direct PKD3–Hsp90 (and PKD2–Hsp90) interaction was detected by PLA experiments in LNCaP cells, demonstrating that Hsp90 also chaperones PKD3 and PKD2 in non-metastatic prostate cancer cells (Figure S5). To address the direct output of PKD3 on cell migration, we transfected LNCaP cells with a PKD3 construct fused to GFP using GFP as control (Figure 5E). The efficacy of gene transfection using EGFP and EGFP–PKD3, and the solubility of PKD3 were confirmed by Western blotting and fluorescence microscopy (Figure S6A,B). PKD3 overexpression increased the migration of LNCaP cells by 34%, showing that PKD3 per se promotes invasive behavior (Figure 5F). Hsp90 inhibition by GS significantly attenuated cell motility in EGFP transfected cells, suggesting Hsp90-dependent migratory processes. Moreover, GS completely abolished the increased migration of EGFP–PKD3 overexpressing LNCaP cells showing that PKD3 migratory activity entirely depends on Hsp90 in this cell line (Figure 5E,F). Thus, PKD3 expression enhances cell invasion, but only when its stability is ensured by the Hsp90 chaperone.

## 4. Discussion

**Figure 6:**
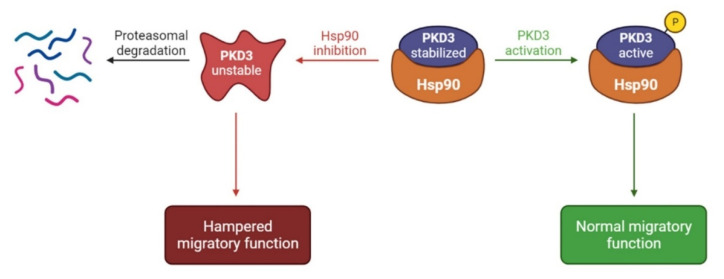
A model for the Hsp90–PKD3 interaction. Hsp90 binds and stabilizes the partially folded, unstable PKD3, probably facilitating regulatory interactions and posttranslational modifications. Phosphorylated and activated PKD3 might still reversibly bind to Hsp90 and exert its cellular functions, such as inducing the Ser536 phosphorylation of NF-κB p65. Hsp90 depletion by pharmacological or genetic interventions or by proteotoxic stress triggers PKD3 destabilization, which attenuates cell migration and other PKD3-mediated functions. Unstable PKD3 undergoes proteasomal degradation. Please note this model is confined to the role of Hsp90 and does not include other mechanisms that regulate PKD3 activity.

The molecular mechanisms underlying prostate cancer metastasis are still elusive. In this study, using two castration-resistant prostate cancer cell lines, we identified PKD3 as a novel Hsp90 client and showed that this interaction contributes to prostate cancer cell migration (Figure 6).

It was reported that PKD2 and PKD3 positively regulate cell migration [10,36]. We observed that CRT0066101, an effective and widely used pan-PKD inhibitor both in vitro and in vivo, attenuated cell migration in doses comparable to those applied in other studies. Since CRT0066101 is not an isoform-specific PKD inhibitor, and it might effectively inhibit other proteins implicated in cell migration, we performed a specific PKD3 knockdown by siRNA. In accordance with the literature, PKD3-silenced prostate cancer cells showed decreased migratory ability [15]. Intriguingly, comparing the sensitivity of DU145 and PC3 cells towards CRT0066101 or ganetespib, PC3 cells responded more readily to both inhibitors. However, this difference disappeared after PKD3 silencing. The motility of both cell lines relies on PKD3 at the same degree, which was confirmed by the ineffectiveness of PKD3 overexpression in PC3 cells. The higher anti-migratory effect of CRT0066101 compared to PKD3 knockdown might be explained by a presumable off-target effect of the inhibitor or an effect mediated by PKD2. Together with the increased migration of low metastatic LNCaP cells upon ectopic PKD3 expression, it suggests that an optimal PKD3 activity promotes prostate cancer cell migration.

Hsp90 constitutes 1–3% of the total cellular protein, which increases two-to-tenfold in tumor cells to fulfill an increased demand to stabilize the oncogenic proteome in stressful tumor microenvironment [22,23]. Through stabilizing several clients, Hsp90 also participates in the regulation of tumor cell migration and metastasis [22,27,35,37,38]. Confirming a recent study, we demonstrated that pharmacological Hsp90 inhibition by the clinically investigated ganetespib compromised androgen-independent prostate cancer cell migration [37]. We found that ganetespib dose-dependently depleted the protein level of PKD3, PKD2, and Akt, which are important regulators of prostate cancer progression [20,32]. Our further findings using PLA, co-immunoprecipitation, and proteasome inhibition show that a direct interaction with Hsp90 stabilizes PKD3 conformation. The fact that the PKD3–Hsp90 interaction exists in non-metastatic LNCaP cells suggests this interaction is a fundamental requirement for PKD3 stability independently of the invasive nature of prostate cells.

Previously, in a system-wide analysis, PKD2 was identified as an Hsp90 interacting protein [19]. Later, in another study, Azoitei et al., using different cancer cell lines, confirmed and characterized the role of the Hsp90–PKD2 chaperone–client interaction in tumor survival and angiogenesis [20]. Hsp90 binds kinases through their kinase domain [18,19]. As the catalytic domains of the human PKD isoforms show over 90% sequence homology [39], it might be tempting to conclude that the Hsp90–PKD3 interaction is a direct consequence of high structural similarity to PKD2. However, despite intense research, no specific Hsp90-binding motif or domain was identified and different kinase (e.g., ErbB and Raf) isoforms exhibiting high sequence homology greatly diverge in their Hsp90-dependence [18]. Moreover, even single amino acid changes at various regions of oncogenic kinase mutants (such as EGFR L858R or BRAF V600E) render them both unstable and Hsp90-dependent. Finally, a systematic analysis of the Hsp90 clientele revealed that instead of particular amino acid sequences, Hsp90 binding is solely determined by the thermodynamic instability of the protein [19]. These facts, together with our findings, provide evidence that the conformational instability of PKD3 is not a consequence of its homology to PKD2. Thus, PKD3 is a novel Hsp90 client protein.

Hsp90 not only stabilizes the native conformation of proteins, but also affects their interaction with their partners. Notably, several Hsp90 clients interact with PKD3, among both PKD3-regulated downstream (i.e.,: Akt, Erk1/2, MMPs [12,14,40]), and PKD3-activating upstream proteins (i.e.,: PLC/PKC [41]). This might be one plausible, although not exclusive, explanation to the greater efficacy of Hsp90 inhibition compared to PKD inhibition. Alternatively, it may be due to the inactivation of other cell migration promoting Hsp90 clients. In more than half of the tumors, Hsp90 has been shown to form a superchaperone-complex with other chaperones and co-chaperones, called the epichaperome [42]. This compact protein network facilitates tumor survival, but at the same time appears to possess a higher sensitivity to Hsp90 inhibition [42,43]. We hypothesize that this molecular mechanism might be extended to Hsp90 complexes promoting cancer cell migration, which may stabilize various clients including PKD3. It may provide a favorable single-hit multi-target therapeutic response in metastatic prostate cancer.

The observation that PKD3 silencing did not augment the effect of Hsp90 inhibition on cell migration indicates that the migration-promoting function of PKD3 entirely depends on Hsp90 in DU145 and PC3 cells. Moreover, ectopic PKD3 expression enhanced the migration of the weakly metastatic LNCaP prostate cancer cells, but only when the protein’s stability was ensured by the Hsp90 chaperone. These results confirm that the stabilization of PKD3 by Hsp90 is essential to promote cell migration (Figure 6). We note that Hsp90 is not the only regulator of PKD3. The mechanisms of upregulation of PKD3 protein level in metastatic prostate cancers are yet unclear. At the post-translational level, PKD3 is activated by protein kinase C mediated phosphorylation in various, including prostate tissues [9]. Besides, RhoA proteins activate PKD3, which executes an inactivating phosphorylation on SSH1L and simultaneously activates PAK4 and LIMK thereby directly promotes cell migration [10]. However, the Hsp90-dependent conformational stabilization is fundamental as it directly regulates the amount of activation-competent PKD3 protein level. Besides, this mechanism provides a scaffold and enables various other protein-protein interactions and regulatory events to take place.

PKD3 exerts its positive regulatory effect on cell migration through multiple mechanisms involving regulation of the cofilin cycle, phosphorylation of NF-κB and GIT1, and deactivation of HDAC1 [10,15,44]. We showed that Hsp90 regulates the PKD3-dependent Ser536 phosphorylation of NF-κB p65. The phosphorylation of Ser536 by PKD3 as well as that of Ser276 by PKD2 are both critical for the transactivation of NF-κB and the expression of the urokinase-type plasminogen activator (uPA), which is in turn, indispensable for prostate cancer cell invasion [15]. As PKD3 and PKD2 are both Hsp90 clients ([20] and our study), it is plausible that their shared activities in prostate cancer metastasis are co-regulated by Hsp90.

Although our experiments examined cell migration toward a chemotactic agent, PKD3 is implicated in cell invasion and endothelial-to-mesenchymal transition (EMT) as well through induction of matrix metalloproteinases (MMP-1, MMP-9, and MMP-13) [14,40]. Notably, extracellular Hsp90 chaperones MMP-2, MMP-3, MMP-7, and MMP-9 suggesting a higher overlap between the migratory function of Hsp90 and PKD3 [38,45,46]. Beside cell migration and invasion, PKD3 is also implicated in prostate cancer cell survival through Akt and Erk1/2 and in angiogenesis through mast cell recruitment [12,47]. Furthermore, it initiates cell proliferation in breast cancer through mTORC1-S6 kinase 1, Erk1/c-Myc axis, Hsp27, and HDAC4/5/7, and it positively regulates the immune suppressor PDL1 in oral squamous cell carcinoma (OSCC) [48,49,50,51]. We speculate that the Hsp90–PKD3 interaction may not be restricted to prostate cancer, but exists in other tumors as well. Likewise, it might not only promote cancer cell migration and invasion, but also proliferation and survival. These speculations might be relevant subjects of further studies.

What might be the physiological outcome of PKD3 stabilization in normal tissues? In hepatocytes, PKD3, the dominant PKD isoform, suppresses insulin signaling and provides negative feedback for cholesterol and triglyceride synthesis, and its overexpression causes insulin resistance [52]. In cardiomyocytes, PKD3 induces pathological cardiac hypertrophy via expression of hypertrophic transcription factors [53]. Based on the abovementioned facts, it seems that the tissue-specific disruption of Hsp90–PKD3 complex by pharmacological agents might be a potential therapeutic approach not only in tumors, but in other metabolic and cardiovascular diseases. In contrast, PKD3 deletion in mice drives liver fibrosis through macrophage activation [54]. Furthermore, during late embryonic development, PKD3 protein expression increases in the central nervous system and cardiac and skeletal muscle tissues, and remains high in adulthood [55]. In these postmitotic tissues, the capacity of Hsp90 might limit pivotal functions of PKD3. Chaperone capacity might decline temporarily under stress [31,56] and progressively during aging [57,58]. Hence, boosting the capacity of Hsp90 and co-chaperones by HSF1 activators [59] might be a therapeutic intervention for the facilitation of PKD3 function through its stabilization. Likewise, the disruption of Hsp90-PKD3 complex by Hsp90 inhibitors may be a double-edge sword especially in multi-morbid cancer patients. The tissue-, chaperone-capacity-, and client-specific interventions lay out potential directions for future research.

Taken together, our study identifies a chaperone–client relationship between Hsp90 and PKD3 in prostate cancer cells, which is essential for PKD3 to exert its cell migratory functions. Our findings provide an insight into the regulation of PKD3 function through its conformational stabilization and may contribute to better understand the molecular mechanisms leading to prostate cancer progression.

## Data Availability

The data obtained during this study are included in this article and its Supporting Information.

## Supplementary Materials

The following supporting information can be downloaded at: https://www.mdpi.com/article/10.3390/cells12020212/s1, Figure S1: PKD protein levels in prostate cancer cell lines. Figure S2: Anti-PKD3 siRNA treatment. Figure S3: Effect of Hsp90 inhibition on the Hsp90–PKD3 and Hsp90–PKD2 interaction. Figure S4: Effect of ectopic PKD3 expression on PC3 cell migration. Figure S5: Hsp90 forms a direct complex with PKD3 in LNCaP cells. Figure S6: Ectopic PKD3 expression in LNCaP cells.
