# Supplementary material for: Protein Kinase D3 (PKD3) Requires Hsp90 for Stability and Promotion of Prostate Cancer Cell Migration
Source: Cells. 2023 Jan 4;12(2):212. doi: 10.3390/cells12020212 (PMC9857065; doi:10.3390/cells12020212)
Supplement: Supplementary file 1 [file cells-12-00212-s001.zip › cells-1969032-supplementary.pdf]

Supporting Information

Protein kinase D3 (PKD3) requires Hsp90 for stability and promotion of prostate cancer cell migration

Attila Varga[1,2*], Minh Tu Nguyen[1], Kinga Pénzes[1,2⁋], Bence Bátai[1,2#], Pál Gyulavári[1,2§], Bianka Gurbi[1,2], József Murányi[1,2], Péter Csermely[1], Miklós Csala[1,2], Tibor Vántus[1,2], Csaba Sőti[1*]

Content:

- **Figure S1:** PKD protein levels in prostate cancer cell lines.
- **Figure S2:** Anti-PKD3 siRNA treatment.
- **Figure S3:** Effect of Hsp90 inhibition on the Hsp90-PKD3 and Hsp90-PKD2 interaction.
- **Figure S4:** Effect of ectopic PKD3 expression on PC3 cell migration.
- **Figure S5:** Hsp90 forms a direct complex with PKD3 in LNCaP cells.
- **Figure S6:** Ectopic PKD3 expression in LNCaP cells.

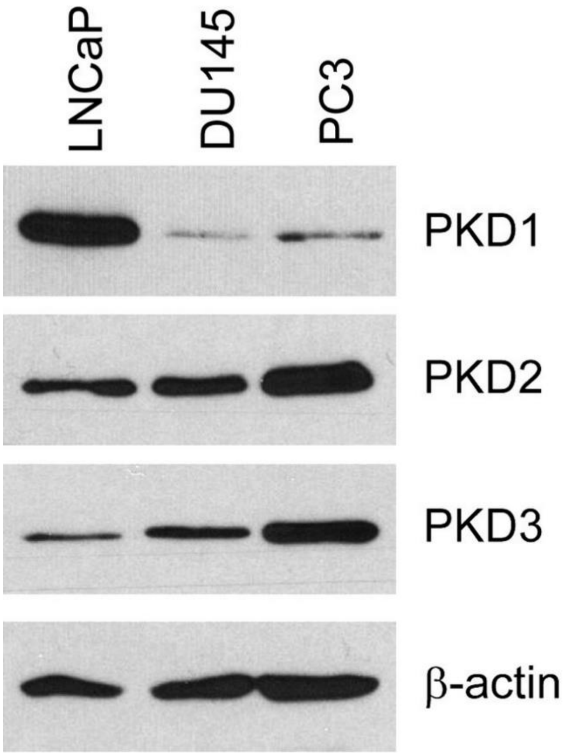

Figure S1. PKD protein levels in prostate cancer cell lines. A representative Western blot from three independent experiments showing PKD protein isoform profiles of the androgen-dependent low metastatic LNCaP and androgen-independent high metastatic DU145 and PC3 prostate cancer cell lines employed in this study. Note the PKD1-PKD3 switch between LNCaP and especially PC3 as well as the higher PKD2 and PKD3 protein levels of PC3 compared to DU145 cells.

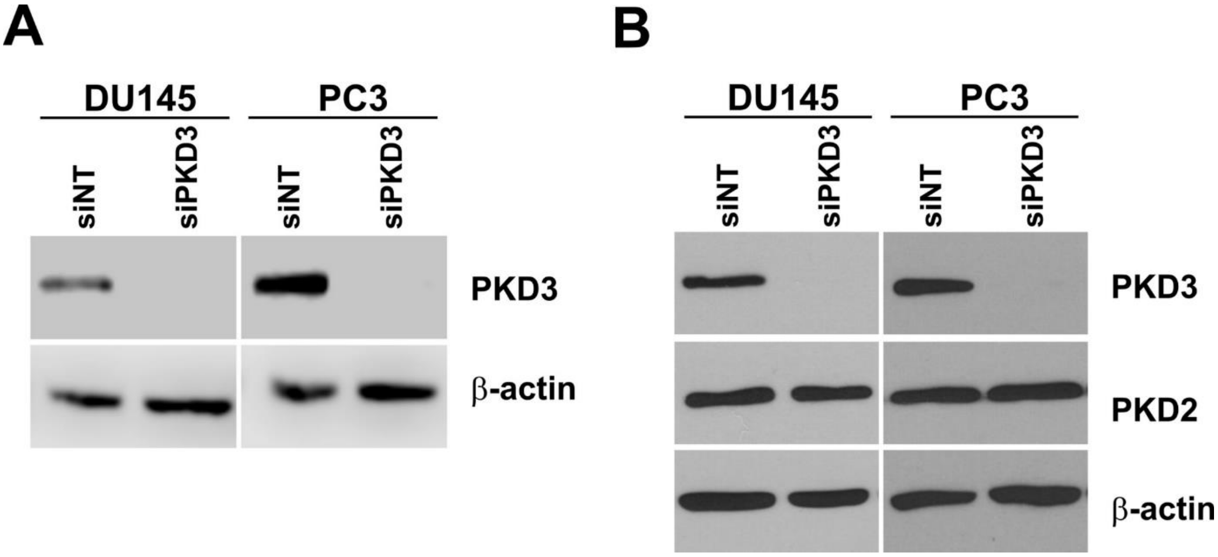

Figure S2. Anti-PKD3 siRNA treatment. **(A)** A representative Western blot showing PKD3 gene silencing by siRNA at the cell migration experimental conditions in DU145 and PC3 cells. **(B)** In a separate experiment, we demonstrated that the anti-PKD3 siRNA did not interfere with PKD2 in these cell lines.

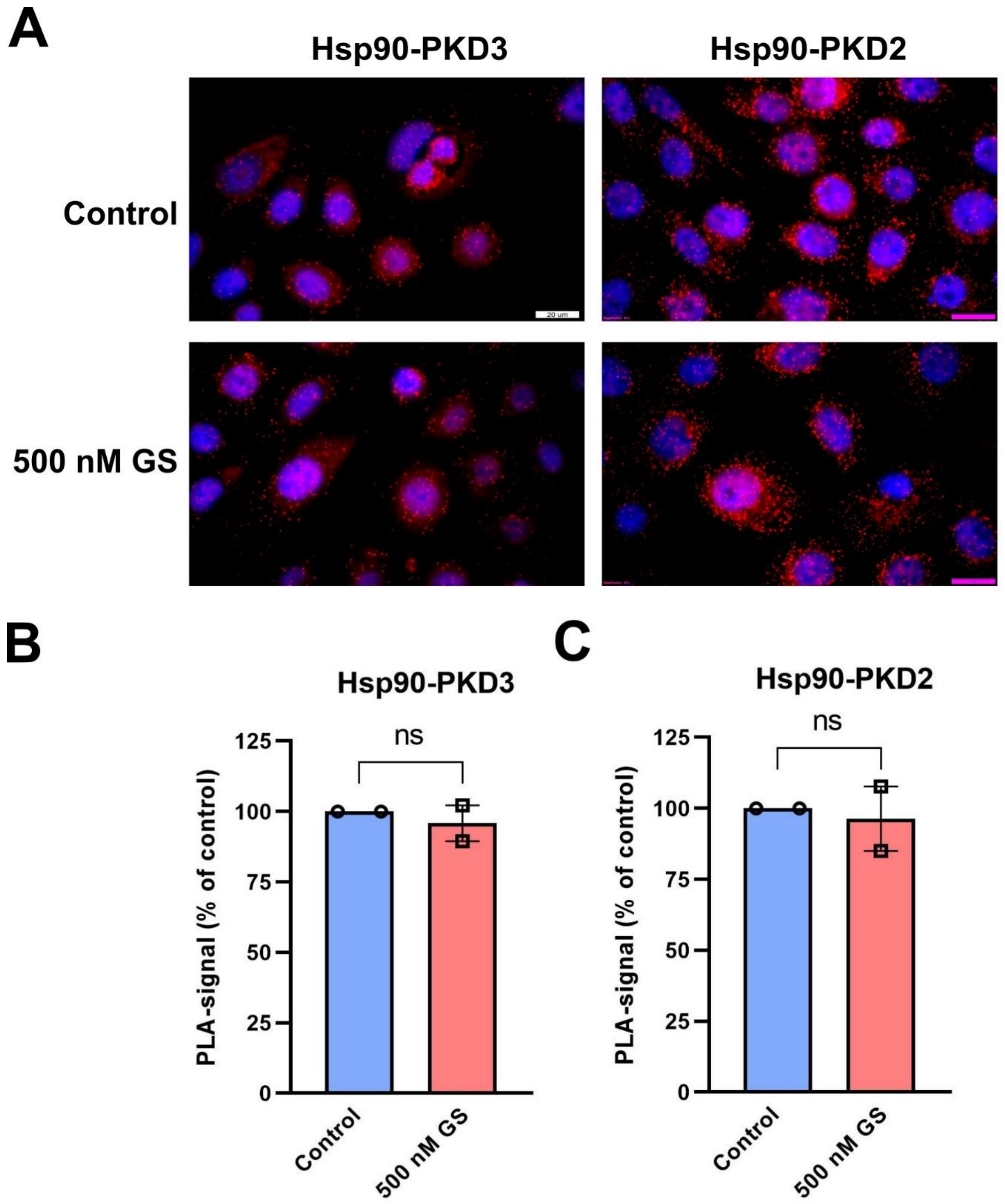

Figure S3. Effect of Hsp90 inhibition on the Hsp90-PKD3 and Hsp90-PKD2 interaction. PC3 cells were treated with 500 nM GS for 4 hours, then PLA-assay was performed. **(A)** Cells were probed with the indicated antibodies to detect direct protein-protein interactions *in situ*. Scale bars represent 20 µm. Images are representatives of two independent experiments. Quantification of the effect of GS on the interaction of Hsp90 with PKD3 **(B)** and PKD2 **(C)**.

Five photos were taken from every sample and dots from five cells from every photo were counted. Graphs represent mean ± SEM.

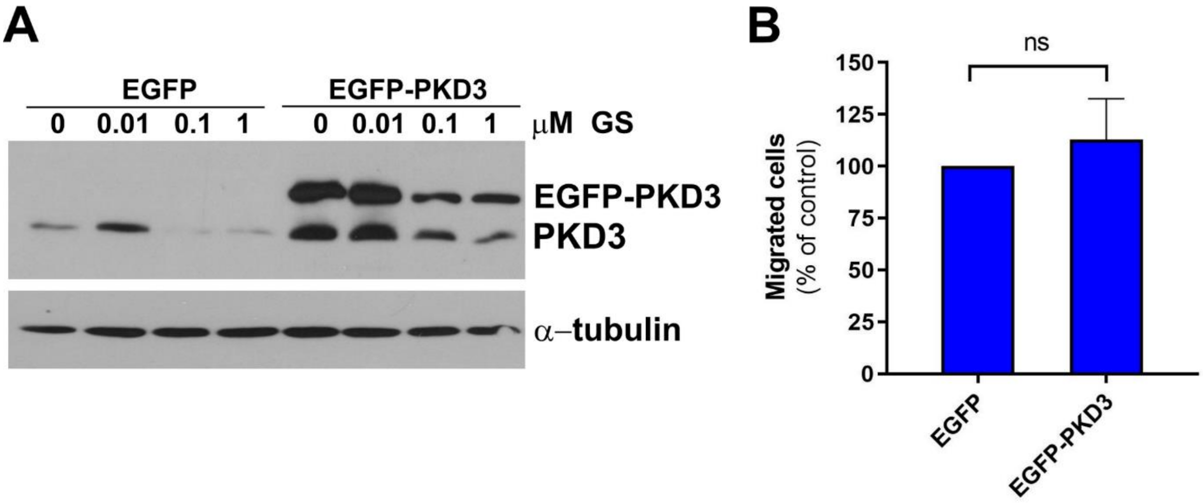

Figure S4. Effect of ectopic PKD3 expression on PC3 cell migration. **(A)** A representative Western blot showing the overexpression and depletion of ectopic EGFP-PKD3 upon a 48-hr GS treatment in PC3 cells. Images are representatives of two experiments. Ectopic expression of EGFP-PKD3 did not enhance the migration of PC3 cells. Data (mean ± SEM) were calculated from 3 independent experiments.

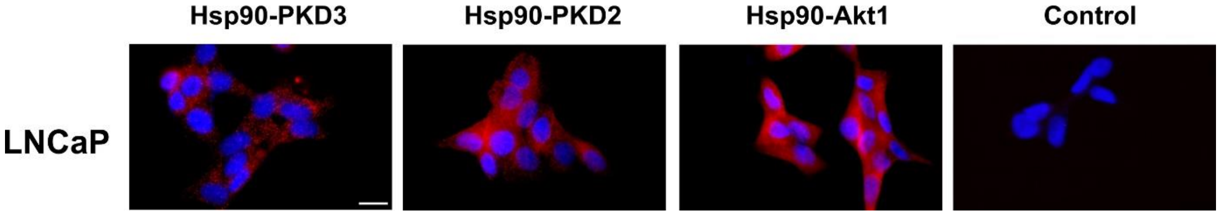

Figure S5. Hsp90 forms a direct complex with PKD3 in LNCaP cells. Cells were probed with the indicated antibodies to detect direct protein-protein interactions *in situ* applying PLA. Controls contained anti-mouse and anti-rabbit secondary antibodies. Scale bars represent 20 μm. Images are representatives of three independent experiments.

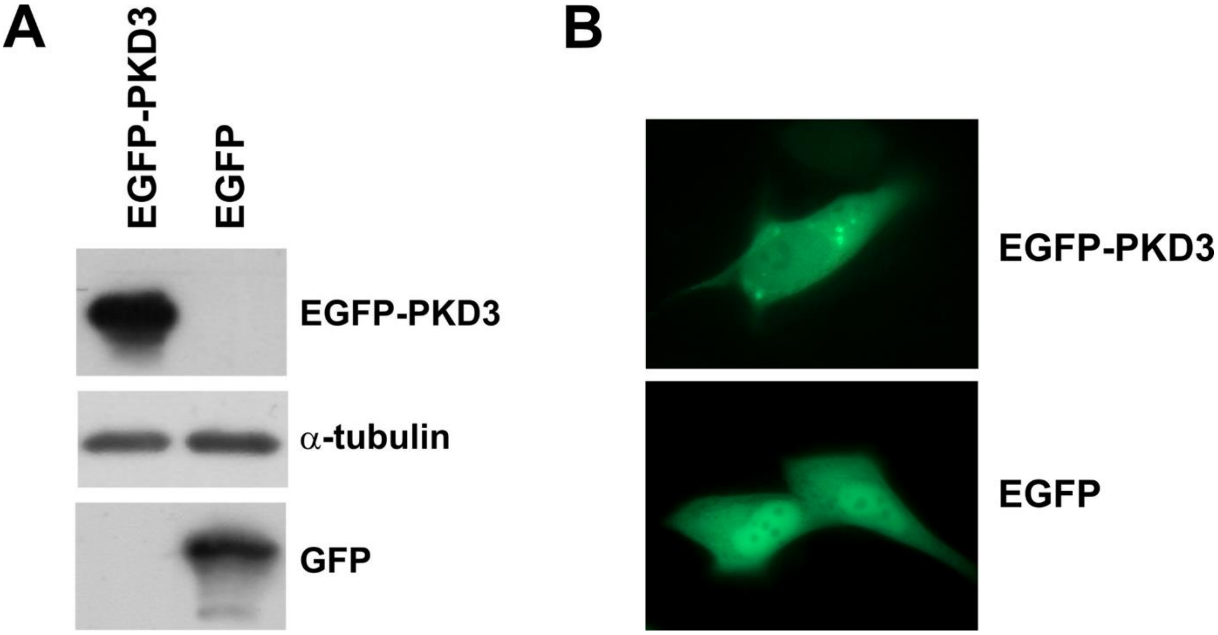

Figure S6. Ectopic PKD3 expression in LNCaP cells. A representative Western blot (**A)** and fluorescence microscopy image **(B)** showing the efficacy of EGFP and EGFP-PKD3 gene transfection and localization of the overexpressed proteins in LNCaP cells.
